# New reporter cell clones to determine the biological activity of human type I interferons

**DOI:** 10.1186/1753-6561-5-S8-P4

**Published:** 2011-11-22

**Authors:** Milagros Bürgi, Claudio Prieto, Marcos Oggero, Mariela Bollati-Fogolín, Marina Etcheverrigaray, Ricardo Kratje

**Affiliations:** 1 Cell Culture Laboratory, School of Biochemistry and Biological Sciences, Universidad Nacional del Litoral. Ciudad Universitaria – C.C. 242 – (S3000ZAA) Santa Fe, Provincia de Santa Fe, Argentina; 2 Cell Biology Unit, Institut Pasteur de Montevideo. Mataojo 2020 (11400) Montevideo, Uruguay

## Background

Interferons (IFNs) are potent biologically active proteins synthesized and secreted by somatic cells of all mammalian species. They play an important role in the immune response and defence against viruses because they have antiproliferative, antiviral and immunomodulatory activities [1]. They are widely used as biopharmaceuticals, so their potency must be correctly identified. Usually, the biological activity is quantified by a bioassay based on its capacity to induce an antiviral state in target cells. Antiviral assays may be subject to high inter- and intra-assay variations having the drawbacks of using viruses [2]. Therefore, a set a new human cell lines derived from different tissues were developed using the enhanced green fluorescent protein (eGFP) gene under the control of type I IFN-inducible Mx2 promoter. In addition, having reporter gene assays derived from cells of different tissue origins would allow us to design studies aiming to evaluate how IFNs induce their actions through the human body.

## Results

Three human tissue-derived cell lines: A549 (lung cancer cells), HEp-2 (epidermoid larynx carcinoma cells) and HeLa (cervical adenocarcinoma cells) were used to develop new reporter gene assays based on the expression of the eGFP gene under the control of type I IFN-inducible Mx2 promoter.

Six stably transfected Mx2/eGFP lines (two of each wild type cell line) were obtained and selected during 10 days with the antibiotic neomycin.

**Figure 1:**
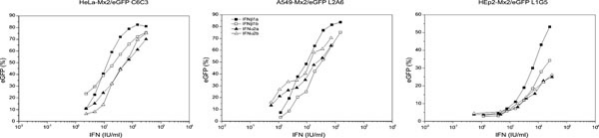
Reporter gene assays based on the eGFP gene expression after the induction of Mx2 promoter by type I IFNs.

The Mx promoter sequence, cloned upstream to the eGFP, responded specifically and quantitatively to type I IFNs (IFN-α and IFN-β) using the new reporter cell lines (Figure 1). Therefore, the three cell lines showed that eGFP percentage (measured by flow cytometry) rose as IFN increased, reaching maximum values of 25-85% activated cells, depending on the cell lines and the type of IFNs. No eGFP expression was observed for negative controls, indicating that there was no basal expression of the reporter protein.

Two subtypes of IFN-α and IFN-β were assayed: the *E. coli*-derived IFNs-α having the amino acid position 23 occupied by the residue Lys (IFN-α2a) or by the residue Arg (IFN-α2b) and the *E.coli*-derived IFN-β with the residue Cys 17 replaced by Ser (IFN-β1b) or the glycosylated CHO cell-derived IFN-β (IFN-β1a). The dose-response relationships corresponding to both IFN-β subtypes showed higher slopes (43 ± 5 IU/ml) than those of IFN-α subtypes (23 ± 12 IU/ml). Considering that type-I IFNs act through a common receptor complex (ifnar) and that mutational analysis revealed distinct binding sites for these IFNs on ifnar [3], the above mentioned results give more evidences that differences in IFN α/β recognition may be associated with cytoplasmic signaling.

Analyzing in more detail the linear dose-response relationships for each cell line (Table 1), A549 cells showed the lowest detection limit for all IFN subtypes (0.24 – 0.48 IU/ml). Comparing IFN-α subtypes, no differences in detection limit were observed using any of the cell lines. Contrarily, differences in responses were observed when comparing the activity of IFN-β subtypes in HEp cell lines. Therefore, the standardization of several assays to measure the potency of IFNs might be carried out using the cell lines herein documented.

**Table 1:** Linear dose-response relationships from reporter gene assays employing different human cell lines.

IFN	HEp L1D7	HEp L1G5	HeLa C5G6	HeLa C6C3	A549L2A6	A549L2G9
**IFN-α2a**	1.52 – 390	12.20 – 25,000	7.8 – 1,000	1.95 – 500	0.24 – 16	0.24 – 125
**IFN-α2b**	0.76 – 390	12.20 – 50,000	7.8 – 1,000	1.95 – 500	0.24 – 4	0.24 – 8
**IFN-β1a**	0.05 – 200	3.05 – 6,250	3.9 – 250	1.95 – 31.25	0.24 – 32	0.48 – 62
**IFN-β1b**	3.05 – 200	12.20 – 50,000	3.9 – 1,000	3.90 – 500	0.24 – 32	0.48 – 62

## Conclusions

These systems have several advantages when compared to antiviral activity assays and other reporter gene systems: they can determine the potency of type I IFNs using only one cell line; they are very fast having the specificity of the Mx promoter. Also, they are sensitive and safe, showing reproducible responses in a dose-dependent manner. Outstandingly, the main contribution of this work was the development of alternative reporter systems as suitable candidates to evaluate the way that IFNs induce their activity in different human tissues.
